# The Occurrence of Laryngeal Penetration and Aspiration in Patients with Glottal Closure Insufficiency

**DOI:** 10.1155/2014/587945

**Published:** 2014-03-11

**Authors:** Ali Rajaei, Ebrahim Barzegar Bafrooei, Fariba Mojiri, Mohammad Hussein Nilforoush

**Affiliations:** ^1^Speech and Language Pathologist, Musculoskeletal Research Center, Isfahan University of Medical Sciences, Isfahan, Iran; ^2^Audiology Department, School of Rehabilitation Sciences, Isfahan University of Medical Sciences, Isfahan, Iran

## Abstract

Glottal closure during the pharyngeal phase of swallowing is one of the important steps in protecting the airway. Generally, it is believed that any deficiency in this process can lead to laryngeal penetration and aspiration. This study investigated the incidence of laryngeal penetration and aspiration among 44 patients with glottal closure insufficiencies that were referred for voice and swallowing evaluation to our institution. The videostroboscopy and 3 oz water swallow test were performed for all of the patients and dysphagic patients were screened and referred for videofluoroscopy. Overall, 15.90% of patients demonstrated signs of laryngeal penetration (13.63%) and aspiration (2.27%). The patients with the pattern of incomplete closure illustrated the highest percentage of penetration-aspiration (21.73%, 4.34%) among other GCI patterns. Thus, early interventions for these patients' swallowing condition seem necessary.

## 1. Introduction

**Figure 1:**
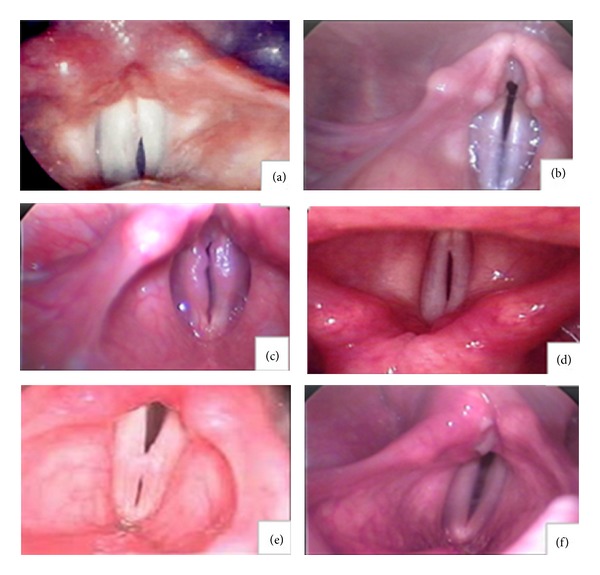
Different pattern of GCI. Different patterns of glottal closure insufficiency (GCI): (a) anterior chink, (b) posterior chink, (c) irregular, (d) spindle (e), hourglass, and (f) incomplete closure.

While swallowing is one of the most critical requirements of every human, the affected swallow could be the source of pain, nutritional incompetency, and loss of health [1]. Any deficiency in the safety of swallowing can lead to laryngeal penetration and aspiration, and these outcomes can result in adverse health consequences such as pneumonia and even death [2, 3]. One of the protective mechanisms in the swallowing process is glottal closure. Laryngeal closure will initiate with arytenoids adduction, glottal closure, and respiratory apnea and will be accompanied with laryngeal elevation and epiglottis inversion [4, 5]. One of the issues that can lead to incomplete airway closure during swallowing is glottal closure insufficiency (GCI). Belafsky and his colleagues defined GCI as a “form of laryngeal hypofunction during which the closed phase of phonation, which is normally 50% of the cycle of vibration, is 45% or less” [6]. With regard to the form of glottis closure, GCI can be observed in one of the six configurations: (I) anterior chink, (II) posterior chink, (III) irregular, (IV) spindle, (V) hourglass, and (VII) incomplete closure (Figure 1) [7].

According to Colton study, “An anteriorly remarkable opening of the vocal folds is named anterior chink. If several contact points with openings in between exist along the vocal folds, it refers to irregular. When the folds close posteriorly and anteriorly except in midsection area, the pattern is bowing. A posteriorly opening in the vocal folds refers to posterior chink. Considerable narrowing of opening at about vocal fold's midpoint is named hourglass. If any portion of the folds do not touch each other, the pattern is incomplete closure” [8]. Symptoms of GCI are inconsistent and can include inconsistent dysphonia, effortful speaking, vocal fatigue, diplophonia, and dysphagia [7]. Laryngeal penetration and aspiration are usually evident following the GCI and can cause the devastating effects on the quality of life and health issues of affected patients [9–11]. These events typically can be diagnosed by videofluoroscopy and fiberoptic endoscopy. Limited data exists about the incidence or prevalence of laryngeal penetration and aspiration among patients with GCI. The incidence of penetration and aspiration is among patients with glottis insufficiency ranging from 24% to 61% depending upon the different causes in different studies [12–17]. Most of these studies usually have considered the effects of unilateral vocal fold palsy (UVFP) on the swallowing, penetration, and aspiration. Furthermore, some of the studies have addressed the effects of different surgeries on the improvement of penetration and aspiration in the UVFP [9–11]. Since there was no study that clearly investigated the incidence of laryngeal penetration and aspiration among different patterns of GCI, the purpose of the present study was to investigate prospectively the incidence of laryngeal penetration and aspiration in patients with different patterns of GCI.

## 2. Patients and Methods

From May 2011 to December 2011, 98 consecutive patients suffering from voice problems were referred to our institution to undergo laryngeal examination and swallowing function. In order to objectively evaluate the vocal cord motion, videostroboscopy was performed for each patient, respectively. One SLP, who was professional in the field of voice pathology, diagnosed patients with GCI and then determined its types. Furthermore, 44 individuals who had glottal closure insufficiency were briefed about the goals of the study and then completed an informed consent. Descriptive data were collected including age, gender, and aetiological factors (Table 1). 

*The inclusion criteria were as follows:*

1. ability to understand spoken Farsi,
2. ability to tolerate sitting position.

*The exclusion criteria were as follows:*

1. evidence of oral or esophageal dysphagia based on clinical assessment,
2. history of other potential causes of pharyngeal dysphagia,
3. Glasgow Coma Scale (GCS) lower than 15 based on bedside assessment,
4. presence of dysarthria based on clinical assessment.

Then, 3 oz water swallow test was performed for all the patients. Eight individuals who were unable to drink the entire amount showed coughing or choking up to 1 min after completion of 3 oz water swallow test or displayed postswallow wet-hoarse vocal quality [18] and were referred for videofluoroscopy of swallow study (VFSS) examination and the other 36 patients were assumed to have no laryngeal penetration or aspiration because they had no symptoms. As in the pharyngeal dysphagia, the challenging consistency is thin liquid and, as most of our patients complained with high thin liquid volumes, we utilized self-administered 60 cc thin liquid Barium with a cup (the same cup which was used for 3 oz water swallow test) for each patient. Then, the incidence of penetration and aspiration was found, respectively, based on penetration-aspiration scale (PAS) by two trained judges that were blinded to each other's judgments. The PAS is an 8-point scale which determines the competency of swallowing by showing the depth of contrast invasion to the airway during swallowing and also the swallower's response to the bolus; that is, the material is completely expelled, partially expelled, or not expelled [19] (Table 2).

## 3. Measurement of Reliability

Interrater measurement of reliability was calculated for judgments of PAS scores based on 8 present VFSS samples of patients. The first author and a trained student independently analyzed the same 8 video recordings and identified all PAS scores. Mean interrater measurement of reliability percentage was 96% based on the interitem correlation (ICC) matrix.

## 4. Results

Table 1 demonstrates the demographic data of patients. In general, the incidence of laryngeal penetration and aspiration in patients with different patterns of GCI was 15.90% (13.63% for penetration and 2.27% for aspiration) and the mean PAS score for all patients with GCI who undergo VFSS was 2.75. The least occurring pattern was anterior chink. None of our participants showed this pattern. On the other hand, the most frequent pattern of GCI was incomplete closure in which 52% (23 of 44) of our referred patients showed this pattern. The overall incidence of other patterns of GCI has been shown in Table 2. The mean PAS score with regard to the pattern of GCI was also measured. Only 1 (female, 27 years old) out of 5 patients with spindle pattern showed penetration with PAS 2. Finally, incomplete closure showed the highest incidence of laryngeal penetration-aspiration so that 21.73% (5 of 23, mean age 39.4) of patients showed penetration and 4.34% (1 of 23, 80 years old) showed aspiration with mean PAS 2.71 (Tables 3 and 4).

## 5. Discussion and Conclusion

The present study is the first report on the overall incidence of laryngeal penetration and aspiration in patients with different patterns of GCI which was based on referral to our medical institution. The incidence of penetration and aspiration in our samples was 15.90% (13.63% for penetration and 2.27% for aspiration) which is lower than other studies (24% to 61%). It is certainly because of the fact that all of the previous works have considered laryngeal penetration and aspiration events just in unilateral paralysis conditions in which the area of glottal gap in this condition is larger than other patterns. In one study it was shown that mean glottal gap areas for patients with aspiration induced from vocal paralysis were significantly greater than that for the nonaspiration group [12]. Unilateral paralysis of vocal cords is the subcategory of incomplete closure. This pattern was the most occurring pattern among other patterns in our populations and encompassed the highest percentage of laryngeal penetration-aspiration which was 21.73% for laryngeal penetration and 4.34% for aspiration. Therefore, this percentage is comparable with the results of other studies. Swallowing is a highly complex process. Either sensory or motor deficits can lead to dysphagia [20]. Glottal closure is a vital step in the pharyngeal phase of swallowing and protection of the airway. It is logically anticipated that any deficiency in the glottal closure process during the swallowing can increase the risk of penetration and aspiration. We encountered three important issues in our study. First, in some patients in our sample, in spite of the absence of complete glottal closure, during the 3 oz water swallow test we did not observe any signs of penetration or aspiration. There could be some possible explanations for it. (1) Probably the size of glottal gap area during swallowing is very important. The larger the area is, the higher the chance of laryngeal penetration and aspiration will be. For example, in the incomplete closure pattern, the glottal gap area may be significantly larger than the posterior chink. (2) Glottal gap area may also be different between individuals with similar patterns. (3) Laryngeal vestibule closure occurred before bolus arrival at the vestibule and was adequate during swallowing despite the observation of incomplete glottis closure during phonation (on stroboscopy). Other possible explanations are sensory or airway protective reflexes impairments and different glottis closure patterns during swallowing and during phonation. Another interesting finding was that in two patients with similar GCI pattern (here, incomplete closure), the occurrence of penetration and aspiration and subsequently the PAS score was completely different.

Two possible explanations could be proposed for this: (1) the same GCI patterns may have had different etiologies; that is, the incomplete closure can be the result of unilateral vocal fold paresis and muscle tension dysphonia. (2) Commencement of the disorder was different between patients, so, the compensatory maneuvers could be utilized either consciously or unconsciously by the patients with chronic diseases which affect the bolus flow process; that is, the patient learns to utilize the effortful swallow maneuver to improve his swallowing efficiency. Another fascinating finding was about some patients who complained about more coughing during water swallow after beginning of their dysphonia but did not show any laryngeal penetration or aspiration during VFSS. It seems that sometimes liquid consistency can be highly specific in patients with pharyngeal dysphagia. Even the thinnest Barium sulfate is thicker than water and can affect our final results. Another explanation is the effect of multiple swallows in this process. It seems that even very fine incoordination between breath control and swallowing will show itself in repeated swallows with larger volumes such as 120 cc. According to the results of this study, the incomplete pattern was the most occurring pattern among other patterns of GCI and these patients were significantly more at risk for laryngeal penetration and aspiration. So, it is reasonable to hear more complaints about dysphagia in these patients and it necessitates monitoring their swallowing condition more in depth. Larger studies with detailed etiological categories and more participants in each category should be designed. It is also advised that in future studies the compensatory swallowing techniques, which are consciously or unconsciously used by patients with glottal closure insufficiency, will be considered.

*Limitation of Study.* According to results, larger size of cases could be helpful to get the exact outcome and, because of glottal closure insufficiency, some cases could not complete the study.

## Figures and Tables

**Table 1:** Patients demographics.

Total number of patients	44	
Gender	16 (male)	28 (female)

Age		
Mean = 35.43	Mean = 29.1	Mean = 39
Range = 20–80	Range = 20–52	Range = 20–80

*Etiological factors *		
Neurologic		
*N* = 11 (25%)	*N* = 4	*N* = 7
Structural		
*N* = 12 (27.3%)	*N* = 2	*N* = 10
Functional		
*N* = 21 (47.7%)	*N* = 10	*N* = 11

**Table 2:** Penetration-aspiration scale.

Category	Score	Descriptions
No penetration or aspiration	1	Contrast does not enter the airway

Penetration	2	Contrast enters the airway and remains above vocal folds; no residue
	3	Contrast remains above vocal folds; visible residue remains
	4	Contrast contacts vocal folds; no residue
	5	Contrast contacts vocal folds; visible residue remains

Aspiration	6	Contrast passes glottis; no subglottic residue visible
	7	Contrast passes glottis; visible subglottic residue despite patient's response
	8	Contrast passes glottis; visible subglottic residue; absent patient response

**Table 3:** Frequency of different patterns of GCI with their PAS score.

Overall PAS score	2.75			
Pattern of GCI		Patients with penetration	Patients with aspiration	Mean of PAS score

Anterior chink	*N* = 0 (0%)	—	—	—
Posterior chink	*N* = 7 (16%)	*N* = 0 (0%)	*N* = 0 (0%)	1
Irregular	*N* = 1 (2.3%)	*N* = 0 (0%)	*N* = 0 (0%)	1
Spindle	*N* = 5 (11.3%)	*N* = 1 (20%)	*N* = 0 (0%)	2
Hourglass	*N* = 8 (18.2%)	*N* = 0 (0%)	*N* = 0 (0%)	1
Incomplete closure	*N* = 23 (52.2%)	*N* = 5 (21.73%)	*N* = 1 (4.34%) PAS score: 7	2.85 Range: (2-3)

**Table 4:** Patients with incomplete closure pattern who undergo VFSS.

Total number of patients	7						
Gender	Male	Male	Female	Female	Female	Female	Female
Age	32	25	80	51	47	57	42
PAS score (60 cc)	3	2	7	2	2	1	3
	Structural	Structural	Neurologic	Structural	Functional	Structural	Functional
